# Effects of Resistance Training on Sarcopenia Risk Among Healthy Older Adults: A Scoping Review of Physiological Mechanisms

**DOI:** 10.3390/life15050688

**Published:** 2025-04-23

**Authors:** Karuppasamy Govindasamy, Chythra R. Rao, Baskaran Chandrasekaran, Koulla Parpa, Urs Granacher

**Affiliations:** 1 Department of Sports, Recreation and Wellness, Symbiosis International (Deemed University), Hyderabad Campus, Modallaguda (V), Nandigama (M), Rangareddy 509217, Telangana, India; 2 Department of Community Medicine, Kasturba Medical College and Hospitals Manipal, Manipal Academy of Higher Education, Manipal 576104, Karnataka, India; chythra.raj@manipal.edu; 3 Department of Exercise and Sports Sciences, Manipal College of Health Professions, Manipal Academy of Higher Education, Manipal 576104, Karnataka, India; baskaran.c@manipal.edu; 4 Faculty of Sport and Exercise Science, UCLan University of Cyprus, Pyla 7080, Cyprus; kparpa@uclan.ac.uk; 5 Department of Sport and Sport Science, Exercise and Human Movement Science, University of Freiburg, 79102 Freiburg, Germany

**Keywords:** resistance training, older, sarcopenia, frailty, physiology, sedentary, sustainable cities

## Abstract

Sarcopenia, characterized by progressive loss of muscle mass and strength, significantly increases health risks in healthy older adults. Resistance training (RT) is believed to counteract sarcopenia through a variety of physiological mechanisms, many of which remain underexplored by public health and physiotherapy professionals. This scoping review aims to consolidate studies that have explored RT programs in mitigating sarcopenia among healthy older adults. A systematic search in four knowledge databases (Web of Science, Scopus, Embase, Cumulative Index for Nursing and Allied Health Sciences Complete) was conducted on 30 April 2024 to consolidate the evidence of RT programs to mitigate sarcopenia risk among healthy older adults. Two reviewers independently screened, consolidated, and synthesized the results based on the Arksey and O’Malley framework. We included 36 studies supporting the RT program for reducing sarcopenia risk among healthy older people. Current evidence, predominantly derived from studies with high selection bias and non-randomized designs, indicates that RT programs may enhance muscle strength in healthy older adults. However, their impact on muscle morphology and mobility appears less pronounced. The dosage and intensity of RT are critical factors influencing these health outcomes. To substantiate the health benefits of RT in healthy older adults and facilitate the translation of research findings into policy-level recommendations, further high-quality, randomized controlled trials are warranted.

## 1. Introduction

Sarcopenia is an age-related muscular disorder characterized by loss of muscle mass and strength, eventually creating difficulty in performing basic and instrumental activities of daily living, such as cooking, climbing stairs, and carrying groceries [1]. Furthermore, older adults with sarcopenia are found to have an increased risk of falls, reduced mobility, and osteoporotic fractures, leading to increased dependency and reduced quality of life [2]. The skeletal muscle loss was found to be 3–8% every decade after 40 years, with accelerated deterioration in muscle strength and mass after 65 years. Further, the cross-sectional area of knee extensors reduced by 16.1% in 12 years from middle working age to the older retirement stage [3]. Maintaining muscle mass, strength, and balance is vital to preserving mobility, preventing falls and cognitive deterioration, and maintaining social engagement and quality of life in healthy older people [4]. Hence, advocating for strategies, including resistance training (RT), to delay progressive senescence-related muscle loss early is crucial in alleviating adverse musculoskeletal events in later life.

Senescence-related muscle atrophy, a classical feature of sarcopenia, is postulated to be shaped by several adverse phenomena, including reduction in satellite cell count and activity, increased heat shock proteins and apoptosis, altered muscle architecture and protein kinetics, reduced muscle fiber and size, altered hormones (insulin and thyroid), dysregulation of cytokine (interleukins, tumor necrosis, and tissue growth factors), and increased oxidative stress and mitochondrial dysfunction, which is further compounded by highly sedentary behaviors [5]. Accumulating evidence now claims that RT can be an effective intervention in mitigating, or at least delaying, these putative mechanisms that underpin sarcopenia in middle and older age [6]. The potential mechanisms through which RT programs prevent sarcopenia may include, but are not limited to, optimized neuromuscular metabolism, regulation of oxidative stress and inflammation, and hormones, such as growth hormone, thyroid hormones, adiponectin, and insulin-like growth factors [1,7,8,9,10]. Along with physiological effects, RT programs are also claimed to improve mental health in the elderly population with or without sarcopenia [11,12]. Both the physiological and psychological benefits of RT ultimately lead to enhanced functional capacity and quality of life, a finding now irrefutably supported by most contemporary empirical studies [13].

Although compelling evidence exists to support the RT program as a countermeasure against sarcopenia, the uptake of RT programs among healthy older adults remains low. A significant barrier to implementation is the lack of awareness about the protective effects of RT against sarcopenia, compounded by challenges in translating research into practice and limited knowledge of RT implementation in low-resource settings, such as homes with less access to gyms. This scoping review aimed to examine the evidence demonstrating the potential physiological effects of RT programs and hypothetical inter-linkages in mitigating sarcopenia and the practical implementation of RT in healthy older individuals. The findings from this review may help public health experts design and implement effective RT programs to combat sarcopenia and improve the quality of life of healthy older adults.

## 2. Materials and Methods

The present scoping review aimed to consolidate the existing evidence that investigates the physiological effects of RT on sarcopenia risks among healthy older adults. The manuscript was reported according to the guidelines of the Preferred Reporting Items for Systematic Reviews and Meta-Analyses Extension for Scoping Reviews (PRISMA-ScR). The checklist of PRISMA-ScR is provided as Supplementary File S1. We administered the search and included the studies until 30 April 2024.

The research problem examined the evidence and the physiological mechanisms underpinning RT programs in mitigating sarcopenia risk in healthy older adults. First, we collated the evidence that explored RT programs as countermeasures to sarcopenia risk in healthy older adults. Second, we extracted the putative physiological mechanisms underlying the RT programs against sarcopenia risk in community-dwelling or institutionalized healthy older adults.

### 2.1. Information Sources and Search

After consulting with our university librarian, we built a search strategy using the following keywords: RT or strength training, healthy older adults, and sarcopenia risk. We administered the following search strategy: “resistance exercise” OR “resistance exercise training” OR dumbbell OR “barbell training” OR “kettlebell” OR “weight training” OR “calisthenics” OR “resistance bands” AND Sarcopenia OR “muscle loss” OR dynapenia OR frailty”. We administered the search strategy in four electronic databases of peer-reviewed journals, including Embase, CINAHL complete, Scopus, and Web of Science. The search was administered from 27 to 30 April 2024. The search strategy is provided in Supplementary File S2. The retrieved citations were imported to EndNote online (https://www.myendnoteweb.com/EndNoteWeb.html), and duplicates were removed. After de-duplication, two authors shared the folder with the citations and started sorting the studies based on the eligibility criteria mentioned below.

### 2.2. Eligibility Criteria and Source Selection

The eligibility criteria were determined using the PICOS framework (provided in Table 1).

Furthermore, the studies to be included should be published in English, regardless of the year of publication. We excluded studies that administered RT in children, were published in languages other than English, and included RT supplemented by nutrition changes or other concurrent interventions, and protocols and conference proceedings that could not provide contextual information. Two authors (KG and BC) independently screened the studies and met with mutual agreement on the inclusion of the studies.

### 2.3. Data Charting Process and Data Items

A bespoke data charting Excel sheet was prepared to extract succinct content from the studies included for the review. We used a narrative review or descriptive analytical approach to systematically gather contextual and process-oriented data. The charting elements filled into the Excel sheets were the author, year, study design, participant characteristics, context, intervention details (supervised or unsupervised, mode, frequency, duration, intensity, volume, and progression of RT), outcome measures (with a specific focus on markers of sarcopenia risk, such as muscle metabolism, mitochondrial oxidation, oxidative stress, inflammation, hormones, such as growth hormone, thyroid hormones, adiponectin, and insulin-like growth factors, and physical markers, such as functional capacity, muscle strength, mass, and architecture), and critical findings or implications.

### 2.4. Synthesis of Results

We adopted a narrative synthesis of the potential findings of the studies that explored the effects of RT in the prevention of sarcopenia risk among healthy older adults. Further charting of data using tables was administered. Following the narrative discussion, a thematic framework was employed to understand the potential physiological mechanisms through which RT programs may mitigate the risk of sarcopenia and to determine the optimal dose required to combat this risk.

## 3. Results

**Figure 1:**
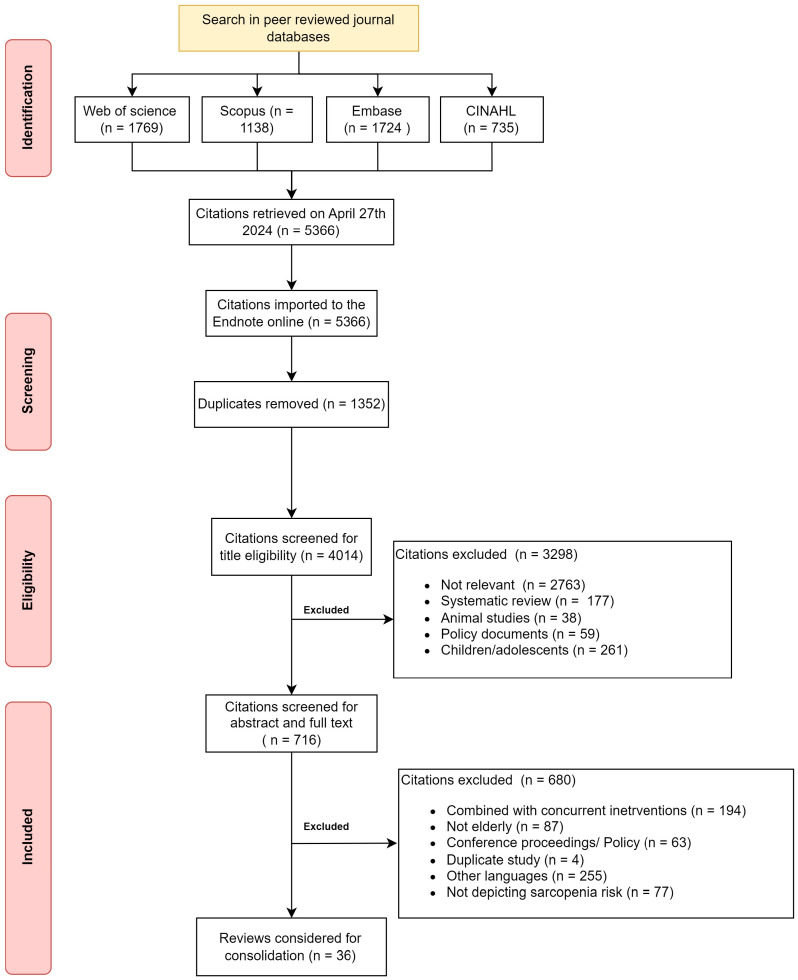
Flowchart depicting citations searched and included in the review.

The initial search yielded 5366 citations from the four databases. After duplicates, 4014 citations were available for screening. The common reasons for exclusion were lack of relevance (69%) and focus on children (6%). After the abstract and full-text screening, the final citations that remained for consolidation were 36 citations available to support the RT program to mitigate sarcopenia in healthy older adults. Figure 1 depicts the screening and inclusion of the citations for the scoping review.

### 3.1. Characteristics of the Included Studies

The characteristics of the included studies are presented in Table 2. Data from each included study were extracted according to the population, interventions, comparator, outcomes, and study design. Most studies were conducted in high-income countries [1,9,14,15,16,17,18,19,20,21,22,23,24,25,26,27,28,29,30,31,32,33,34,35,36,37,38,39,40,41,42,43,44,45,46], with only one study from lower- to middle-income countries (Iran) [47]. The majority of the included studies were randomized controlled trials (n = 22/36, 61%).

### 3.2. Population

A total of 2146 older participants were studied. The majority of the studies recruited both genders, while few specifically recruited sedentary men and women with or without sarcopenia or who were at risk of developing sarcopenia or frailty. Standard criteria for diagnosis of sarcopenia were the appendicular skeletal mass index (males < 7.29, females < 5.93), Short Physical Performance Battery (SPPB) ≤ 8 points score, gait speed in 0 m walk test ≤ 1 m/s, and skeletal mass index ≤ 28% or ≤7.76 kg/m^2^ [15,47]. Few studies involved participants who had sarcopenic obesity with a body mass index of more than 30 kg/m^2^ [22,26,27,29,33,46,47], while few involved institutionalized individuals [25].

### 3.3. Intervention

All of the studies included supervised RT programs. The majority of the studies administered gym-based structured RT programs involving weight plates, hydraulic machines, and barbells in well-equipped gyms [1,9,10,16,20,21,22,23,24,25,28,29,30,31,33,34,35,37,39,40,41,42,43,45,46], while few studies administered body supported exercises, TheraBands, and free weights in the community [14,17,18,26,27,36,38,44,47]. The dose commonly seen among studies is as follows: duration of total intervention—12 weeks (28 days–24 months); intensity and volume: eight exercises for large muscle groups in upper and lower limbs, 1st weeks, 1 set 50% one repetition maximum (1-RM), 2nd week two sets of 60% of 1 RM, 3rd to 12th weeks 75–80% of 1 RM three sets, inter-set rest—60–90 s; and frequency (thrice per week for 12 weeks) using the linear periodization model [30,32,33]. The typical exercise protocol was as follows: Monday and Friday: squats, chest press, lateral pulldown, abdominal crunches, and back extensions; Wednesday: leg extensions, leg curls, chest butterflies, upper back rowing, and calf raises [9,10,39,41]. Few trials were compared based on intensity (low vs. high) [32], speed [42], or periodization (linear vs. undulating) [28]. Community-oriented exercises routinely administered squats, marching on the spot, crunches with body weight, or TheraBands. Only five studies (n = 5/36, 14%) mentioned the standardization of the tasks (passive stretching, meditation, routine care) in the control group [10,25,32,35,36]. Only a few trials successfully progressed the intervention (50–80% 1 RM) for the trial period [22,23,29,31,33].

### 3.4. Outcomes

Almost half of the studies (n = 17/36, 47%) measured the body composition and physical fitness, i.e., handgrip, time-up and test, stair climbing (physical fitness battery), as the primary outcome [1,17,18,22,24,25,27,32,35,36,37,38,40,42,45,46]. Similarly, the subsequent significantly studied outcome was body composition, including lean body mass, fat mass, and appendicular mass indices, through DEXA or bioelectric impedance analysis [10,22,23,24,25,26,27,33,34,39]. A considerable number of studies examined the effects of RT on 1-RM [22,23,29,30], maximum dynamic strength through an isokinetic dynamometer [16,20,23,27,28,30,33,39,43], and peak muscle activation through electromyography [21,28]. Few studies explored the effects on sarcopenic status [32,33,37], Troponin [18], muscle cross-sectional area [28], cognition [29], serum lipids [30], immune–inflammatory markers [insulin-like growth factor IGF-α, T cells and antibodies, C-reactive proteins, CRP, tumor necrosis factor, TNF, interleukins (IL-6, IL-10)] [9,10,14,30,31], reactive oxygen species [1], sleep [30], muscle cross-sectional area through ultrasonogram [1,24], visceral adipose tissue through MRI [23,34], muscle volumes through computer tomography [10,16], bone mineral and fracture risk [47], respiratory functions [32], energy expenditure through accelerometers [36], and behavior change [37,42].

### 3.5. Key Findings

#### 3.5.1. Positive Findings

Almost all of the studies demonstrated favorable effects on physical performance (handgrip strength, time-up and test speed, chair stand time, stair climb ability, wall push) [1,16,17,18,22,27,40,45,46] and muscle strength (1-RM) and power [20,35,36,39]. Furthermore, maximal voluntary force production, peak torque, power, and 1-RM improved in most studies that employed moderate- to high-intensity traditional progressive RT programs [16,22,23,33,37,39,41,45]. Furthermore, the waist–hip ratio [43], lean body mass, and skeletal muscle index improved with a reduction in fat-free mass, which was evident in many studies [9,15,16,24,27,33,34,39]. Muscle growth factors, such as follistatin and myostatin, were found to be influenced by progressive RT programs among healthy older adults [43,44]. Rufino et al., 2023 significantly improved respiratory muscle strength and dynamic lung volumes [32]. Schulte et al. demonstrated a significant improvement in muscle protein synthesis and myostatin–immunoreactive proteins after a 10-week progressive RT program among physically frail older adults [43]. Stair stepping (forward or sides) was found to elicit muscle (vastus lateralis, gluteus maximus, and biceps femoris) activation similar to traditional RT at 60% and 80% 1-RM test [21]. Functional RT (chair standing, stair climbing, cleaning high places) was found adequate to improve executive functions in sarcopenic obese older adults [29]. Traditional progressive RT of 12 weeks improved sleep onset and reduced sleep latency, apneic episodes, and insomnia severity [30]. Further, a few studies demonstrated favorable effects on immune–inflammatory and immune–senescence markers [10,30,31,39]. Vezzoli et al., 2019 demonstrated a significant reduction in reactive oxygen species production after 12 weeks of progressive RT among healthy older adults over 65 years [1]. All of the favorable changes occurred only in the studies that advocated for moderate- to high-intensity progressive RT programs with moderate to high loads, longer durations, and larger volumes [9,10,36,37]. Any additional intervention added to the traditional RT program improved the outcome measures of the intervention added. For example, when added to traditional RT programs, physical activity advice improved activity levels in addition to the regular benefits of RT programs [37].

#### 3.5.2. Null Findings

Few studies found no significant changes in physiological parameters, such as body composition [18], visceral or subcutaneous fat percentages [23], bone mineral density, telopeptides and fracture risk [47], strength or peak force development [42], muscle mass [45], gait speed, physical performance [1,15,25], muscle mass index, quality of life [26], and accelerometer-based physical activity levels [42]. A single-group pre–post designed trial by Perreault did not find any changes in the inflammatory markers after 16 weeks of the RT program [9]. Conversely, Yuenyongchaiwat et al., 2022 found a difference in IL-6 and TNF-α within 12 weeks of RT with elastic bands and pedometer-based aerobic training [14]. Although any systematically organized RT programs brought significant changes in the muscle cross-sectional area, force development, and muscle activation, differences in periodization strategies did not produce any significant differences among the groups [28].

## 4. Discussion

**Figure 2:**
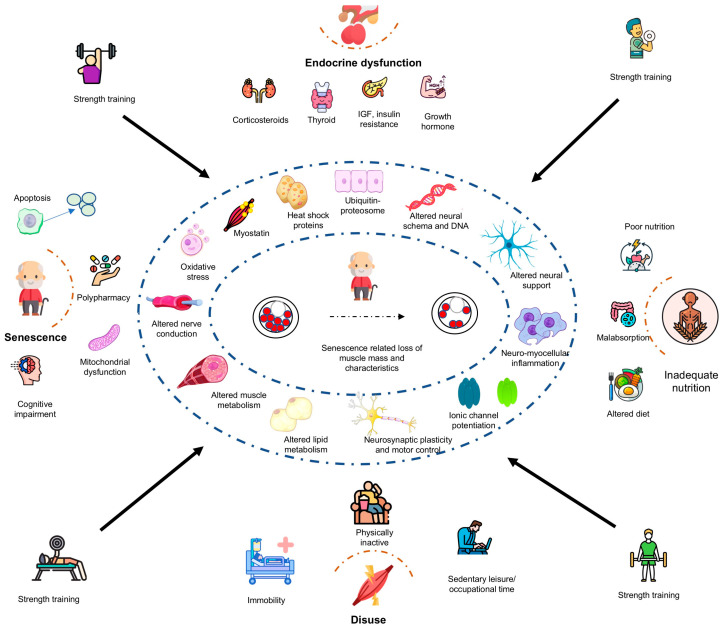
Potential physiological mechanisms underpinning resistance exercise training in the mitigation of sarcopenia risk among healthy older adults. ACE—angiotensin-converting enzyme, APOE—apolipoprotein E, IGF—insulin-like growth factor, IL—interleukin.

The present scoping review explored the physiological effects of RT programs to mitigate the sarcopenia risk in healthy older adults. While substantial evidence suggests that RT programs improve muscle strength, preserve mass, and enhance functional capacity in healthy older adults, their impact on the molecular mechanisms of protein synthesis, muscle breakdown, inflammaging, sleep quality, mental health, and cognitive functions remains inconclusive (Figure 2). Furthermore, the translation of these physiological changes into improved functional capacity, reduced fall risk, and prevention of senescence-related osteoporosis is still being investigated.

While substantial evidence indicates that RT programs can effectively reduce the risk of sarcopenia in healthy older adults [9,10,16,17,20,21,22,24,27,28,29,30,31,32,33,34,35,36,37,38,39,40,41,43,44,46,47,48,49], the majority of trials (n = 13 of 17) did not observe similar favorable effects on muscle mass and biomarkers associated with sarcopenic obesity [1,15,18,23,25,42,45]. This inconsistency may be attributed to variations in intervention characteristics, such as volume, intensity, mode, and frequency, as well as differences in the quantification of outcome measures. A recent systematic review aligns with our findings, indicating that RT has modest effects on both relative and absolute muscle mass but significantly enhances muscle strength in healthy older adults with sarcopenia [50]. The outcomes are influenced by factors like the training period, number of sets, contraction speed, and intensity. As life expectancy increases with the advent of medical advances, healthy older adults in modern society are expected to fulfill several responsibilities, including self-care and being functionally independent in basic and instrumental daily living activities, achieving unrestricted mobility to complete their social roles (taking care of grandchildren, getting groceries), and preventing senescence-related complications (falls and fractures) [51]. RT programs ranging from low- to high-intensity are observed to maintain muscle mass and strength [17,20], thereby offering protection against fall risk and improving physical and social well-being among older adults [21,22].

The majority of trials identified in this scoping review indicated that RT programs have the potential to significantly increase muscle strength and, to a lesser extent, muscle mass in healthy older adults [9,10,25]. Findings from this review are consistent with the meta-analysis provided by Borde et al. (2015), which showed that RT significantly improved muscle strength but had only minor effects on muscle morphology [52]. The meta-regression revealed that the programming parameters of training period, intensity, and total time under tension significantly affected muscle strength. The current literature analysis revealed that RT effects did not or only to a minor extent translate into fall risk reduction, a result that concurs with existing reviews [53]. This finding is also supported by Beijersbergen et al. (2013), who showed only small associations between RT-related improvements in measures of muscle strength, power, and gait speed [53]. Similarly, our review included some studies that investigated RT’s effects on gait speed, which is an important marker of mobility in healthy older adults, and the drawn conclusions remain equivocal [22,25,27,37,46]. More research is needed on the effects of RT on mobility outcomes and fall rates and risk in healthy older adults. Currently, the literature is uniform with regards to RT’s effects on muscle strength, power, and mass. Yet, less is known about the most effective RT methods to improve mobility and reduce fall risks in older adults.

Moreover, RT has been associated with anti-inflammatory effects, which may have implications for cardiovascular disease (CVD) prevention. Sarcopenia and CVD share common inflammatory pathways, including elevated cytokine levels, such as IL-6 and TNF-α, which contribute to vascular dysfunction and metabolic syndrome [54]. Studies indicate that RT reduces systemic inflammation [10,30,55], potentially mitigating CVD risk. By lowering chronic inflammation, RT may serve as a non-pharmacological “polypill” to improve cardiovascular health in sarcopenic populations. A recent systematic review by Momma et al. (2022) demonstrated that RT programs were associated with a 10–20% lower risk of all-cause mortality, cardiovascular disease, total cancer, diabetes, and lung cancer, with the maximum risk reduction observed at approximately 30–60 min per week of performing muscle-strengthening activities [56].

Beyond musculoskeletal benefits, RT plays a role in delaying the onset of chronic dis-eases associated with sarcopenia. Increased muscle mass and strength correlate with both improved insulin sensitivity and upregulation of GLUT-4 transporters, reducing the risk of type 2 diabetes [57]. Furthermore, RT has been shown to influence cancer prognosis by enhancing immune function and reducing systemic inflammation, which is implicated in cancer progression [58]. In this context, Momma et al. (2022) demonstrated in their meta-analysis that RT programs were associated with a reduced risk of total cancer mortality, although dose–response relationships varied across the different cancer types (e.g., colon, kidney, pancreatic, bladder, and lung cancer) [56]. The metabolic improvements associated with RT, including enhanced glucose metabolism and lipid profile regulation, suggest a protective role against metabolic disorders, such as obesity and metabolic syndrome [59].

While increased muscle strength is a direct outcome of RT, its impact on quality of life (QoL) requires further exploration. Ramirez et al. (2018) reported significant QoL improvements in elderly populations following RT programs [40]. However, other studies present mixed results, indicating that the relationship between RT and QoL is not yet fully understood [26,37]. To better understand the impact of RT on QoL, longitudinal studies implementing RT programs in free-living settings are warranted. Although RT is beneficial, its potential risks, particularly for individuals with pre-existing joint conditions, such as osteoarthritis (OA), must be acknowledged. High-intensity RT can exacerbate joint stress, potentially leading to discomfort or injury if not appropriately managed [60]. It is essential to tailor RT programs to accommodate joint limitations by incorporating lower-impact modalities, controlled loading, and progressive overload principles. Studies suggest that supervised RT programs designed with joint health considerations, such as using resistance bands or machines instead of free weights, can mitigate these risks while still providing musculoskeletal benefits [61]. Additionally, proper warm-up, cool-down, and technique correction play a crucial role in preventing joint-related complications in aging individuals.

The evident improvement in physical performance (handgrip strength, time-up and go speed, chair stand time and stair climb ability) may improve daily living and enjoy their social lives [1,17,18,22,45]. Further, sarcopenia prevalence was found to reduce with RT programs, while the control group remained the same [1,32]. Interestingly, few studies reaped success in sleep onset [30], inflammaging status [1], muscle growth factors (follistatin, actinin) [44], anxiety and depression [26] and executive functions [30] among healthy older adults who have undergone classical RT programs. Besides gait speed, an essential determinant of negating roads safely and stair climbing ability, a determinant of negating stairs without inherent fall risks, are found to improve in most of the included studies [22]. Meanwhile, few studies have been conducted to contradict the positive effects of RT programs, primarily on body composition, gait speed, bone density, and muscle mass [18,25,47]. It appears that studies reporting null findings may have implemented low-intensity RT programs, utilizing elastic bands and body weight exercises at suboptimal doses, which may not effectively counteract sarcopenia risk. This could also explain why the majority of these trials did not report adverse events such as falls or cardiovascular incidents during the intervention period. Moreover, the inclusion of studies with small sample sizes and non-randomized designs in this review introduces a significant risk of selection bias, potentially compromising the validity of the findings. Such methodological limitations hinder the ability to draw definitive conclusions regarding the efficacy of RT interventions in healthy older adults.

### 4.1. Dose of Resistance Exercise Program

Based on the available literature, the design and dosage of progressive resistance exercise training, both in the workplace and during off-hours, can be implemented as outlined in Table 3. This guidance may assist exercise professionals and public health experts in developing and implementing appropriate RT programs for healthy older adults to maximize the physiological and health benefits previously discussed.

### 4.2. Caution with Resistance Exercise Training

Resistance testing and training are not without risk among healthy older adults who are healthy with no known disease risk. Exercise-induced muscle damage after acute high-intensity RT may adversely affect the ability to do daily activities and fall risk [62]. Further transient increases in inflammatory markers protein damage may cause transient fatigue and traumatic arthropathies. Hence, appropriate dosing (intensity, duration and frequency) is crucial in preventing muscle injuries, fatigue, and fall risk after RT programs. However, none of the included studies in our review reported adverse events associated with RT programs among healthy older adults.

### 4.3. Limitations

A few limitations of the present review are the following. (1) We administered the search criteria based on our three team members’ knowledge and the librarian’s suggestions. Furthermore, the search was limited to four databases and included studies only published in English. A search of the gray literature and other languages might have provided us with more results. (2) Only a few studies administered RT programs in low-resource community settings. The findings of this scoping review may not be generalizable to low-resource settings. (3) The majority of the studies involved small sample sizes and used heterogeneous methodologies for administering RT (varying in frequency, intensity, and duration), making it difficult to draw conclusive evidence on the effects of RT programs on sarcopenia risk. (4) Despite conducting a systematic search, our scoping review was subject to selection bias. Notably, only three studies with substantial sample sizes (approximately 150 participants each) were identified [33,39,55], while the remaining studies involved smaller cohorts. This raises concerns about small-study bias, as studies with limited sample sizes are more susceptible to overestimating effect sizes and may lack the statistical power necessary for reliable conclusions. Such biases can compromise the validity and generalizability of the findings. This limitation underscores the pressing need for high-quality randomized controlled trials to enable comprehensive systematic reviews that can clarify the effects of RT on sarcopenia risk among healthy older adults. (5) Furthermore, the safety precautions implemented during these trials remain ambiguous, particularly as many employed low-intensity RT programs. While low-intensity RT has been shown to benefit healthy older adults with sarcopenia, the specific safety measures adopted in these studies are often not clearly reported [63].

## 5. Conclusions

RT shows promise as a countermeasure against sarcopenia in healthy older adults by modulating protein catabolism, enhancing muscle growth factors, and mitigating immuno-senescence and inflammaging. Evidence suggests that RT can improve both peripheral and respiratory muscle strength, potentially leading to modest gains in gait speed. However, these findings are constrained by methodological limitations, including small sample sizes, non-randomized study designs, and potential selection biases, which hinder the ability to draw definitive causal inferences. Moreover, the long-term health benefits of RT are influenced by broader factors, such as national policies, peer support, and institutional commitment to implementing RT programs.

## Figures and Tables

**Table 1:** Eligibility criteria of the studies included.

Framework	Inclusion Criteria	Exclusion Criteria
Population	Older adults (≥60 years age) Healthy (i.e., free from chronic diseases that increase frailty)	Middle-aged adults <60 years Patients with chronic diseases (e.g., cardiometabolic diseases and neuromuscular diseases affecting metabolism and mobility)
Intervention	Any RT program > 4 weeks With volume, frequency, duration, and intensity specified Administered in the home or gym Supervised or unsupervised	Exercise training other than RT program Mixed training (RT with aerobic training)
Comparison	Passive control	
Outcomes	Any of the outcomes related to sarcopenia risk Muscle strength Muscle mass Muscle fiber morphology Serum biomarkers (hormones: insulin, leptin, adiponectin; cytokines) Functional mobility tests: time and distance trial test	Psychological outcomes that were not part of the objective
Study design	Intervention trials Randomized controlled trial Non-randomized controlled trial	Observational trials Cross-sectional studies

Abbreviation: RT—resistance training.

**Table 2:** Characteristics of the studies and their key findings.

Author (Year)	Study Design	Country	Participants	Intervention	Outcome Measures	Key Findings
Abreu et al., 2014 [17]	Non-randomized trial	USA	73 participants 34—subset evaluated for troponin	Two interventions: “Peer Exercise Program Promotes Independence” (PEPPI) and “Stay Strong, Stay Healthy” (SSSH) PEPPI: resistance bands, one hr/week, ten weeks SSSH: with and without free weights, ten weeks, twice/week, one hr/session	Senior Fitness Test Handgrip strength TUG Systolic BP Troponin 30 s chair stand Wall push-up Chair sit reach Serum troponin (ELISA kit)	↑ handgrip (+2.1 kg) ↓ TUG (−1 s) ↑ systolic BP (+2 mmHg) ↓ Troponin (−17 pg./mL) ↑ 30 s chair stand (+3) ↑ wall push-up (+3) ↑ chair sit reach (+1 inch)
Adnan et al., 2021 [18]	Non-randomized trial	Malaysia	36 pre-frail, older >Aged 60 years Attending the primary health clinic Frailty index score of 1–2	Developed from “Growing Stronger program/ book.” Body-supported exercises 12 weeks 20 out of 24 sessions Major muscle groups in the upper extremities and lower extremities	12 weeks TUG Systolic BP Troponin 30 s chair stand Wall push-up Chair sit reach	88.9% adherence rate No statistically significant differences Small effect size ↓ TUG time (−0.25 s) ↓ STS duration (−0.41 s, ES = 0.20) ↑ handgrip strength (+0.68)
Aragao-Santos et al., 2019 [20]	Non-randomized controlled trial	Brazil	44 elderly women Aged >60 years >One year of not performing any systematic training Absence of orthopedic/cardiovascular problems	Two interventional (functional FT and traditional TT) and one control group CG FT: 25 min of multipoint muscle of arms and legs, stabilizing spine TT: machines with maximal effort and velocity, 8–12 reps at RPE—7–9 CG: playful multi-joint exercises 12 weeks Thrice weekly, alternative days, 50 min	Maximal dynamic strength and muscle power tests Isometric handgrip test, isometric dead lift test, and muscle endurance	Both interventions increased power, strength, and workability
Baggen et al., 2018 [21]	Non-randomized trial	Belgium	19 healthy elderly women Community-dwelling	IG: forward and lateral stepping exercises RT: traditional resistance exercise (chest press, lateral pull, flies, and straight leg raise) 40, 60, and 80% of 1 RM of congruent RT	EMG of large muscle groups of lower limbs Kinematics: 3-D motion capturing (ViCON)	Gluteus maximus ↑ peak activation matched 60% 1-RM Semitendinosus is not matched Sideways stepping at 30 cm step matches traditional RT
Balachandran et al., 2014 [22]	Non-randomized trial	USA	Local south Miami community 21 sarcopenic, obese adults 60–90 years old Absence of any disease-causing physical limitation	Two intervention groups: circuit and hypertrophy Hypertrophy: 10–12 reps, 3 sets, 70% 1 RM progression, 55–60 min/session Circuit training: 10–12 reps, 3 sets, 50–75% 1 RM, 45 min/session Five lower and six upper body exercises Pneumatic exercise machines 15 weeks	SPPB—physical function Power and strength of upper and lower body Instrumental activities of daily living Body fat % Handgrip strength	Physical function 20% ↑ circuit group (g = 1.1, *p* = 0.02) SPPB ↑ 1.1, ES = 0.6. in circuit training group Normal gait speed ↑ mean difference of 0.3 s (0.09 m/s) in HSC (no change) Lower body power ↑ 158 watts in HSC Leg press peak power ↑ 41% circuit training and 22% in hypertrophy Sit-to-stand ↑ in circuit training group RPE =−1.5 in circuit training group No significant difference in other parameters (6MWD, SMI, grip strength, body fat%)
Banitalebi et al., 2021 [47]	Randomized controlled trial	Iran	63 women >60 years Obese Sarcopenia	Three times per week, 12 weeks Therabands 20 RM Larger muscles (chest, arms, legs) Volume and intensity progressed Warmup 10 min, RT = 60 min, and cool down 10 min 12 weeks	Appendicular lean mass index Fracture risk Bone mineral density Vitamin D and alkaline phosphatase miR-206 and miR-133.	The study findings did not favor RT No change in any of the outcomes RT did not affect serum myomiRs or osteoporosis
Binder et al., 2005 [23]	Randomized controlled trial	USA	91 older adults Sedentary Aged >78 years Physical frailty (physical performance, ADL difficulty, and peak VO2)	IG: three phases of 3 months Phase 1: 22 low-intensity exercises (balance, coordination, flexibility) Phase 2: PRT: six traditional exercises; 1–2 sets of 6–8 repetitions of each exercise at 65% of their 1-RM; progressed to three sets of 8–12 reps/set, 85% 1 RM Phase 3: home ex program for three months CG: low-intensity home exercise program Nine months	MVC—knee extension and flexion—1 RM Body composition—DEXA Visceral subcutaneous adipose tissue (MRI)	Compliance: 140 ± 41 days; average of 2.2 ± 0.3 days/week 1-RM—leg flexion—↑ 17% and leg extension—43% MVC for knee extension↑ (Δ +5.3 ft/lb) Total body FFM ↑ (Δ +0.84 kg) No change in the total trunk, abdominal, or subcutaneous fat (ΔVAT: CG −3.8 ± 29 cm^2^; IG: −7.0 ± 43 cm^2^) Supervised RT ↑ muscle mass in frail individuals
Candow et al., 2011 [24]	Randomized controlled trial	Canada	Twenty healthy older men Aged 64.7 ± 5.0 years Not performing RT for at least 15 months	High-volume supervised RT for 22 weeks (66 sessions) Thrice a week Three sets of 10 RM/set 2 min interest Five upper body, four lower body 70% 1 RM for bench and leg press Machine-based RT	Body fat mass, fat-free mass Muscle girth of upper and lower limb muscles Strength (leg press and bench press 1 RM) At 12 and 22 weeks	Before training, compared to young adults Lean mass ↓ −6 kgs, muscle thickness ↓ −0.6 cm, leg press ↓ −63 kgs, bench press ↓ −47 kgs After 22 weeks of training, IG had muscle mass similar to that of young adults
Cebrià I Iranzo et al., 2018 [25]	Randomized controlled trial	Spain	81 elderly Spanish individuals (>65 yrs.) Institutionalized older Spanish adults with sarcopenia Clinically stable for at least two months	IG: two RT groups: peripheral and respiratory resistance training 5 min warm-up, 20–30 min exercise, 5 min cool down Peripheral training: 10 RT ex, 12 reps/set, 40–60% of maximal isometric strength Respiratory RT: threshold trainer, 7–41 cmH2O Four lower limb and six upper limb, 12 weeks Dropped <80% of the sessions CG: maintain their usual care	Appendicular skeletal mass Knee extensors isometric strength Handgrip strength Ventilatory muscle strength Gait speed At 0 weeks and 2nd and 12th week	No significant changes in muscle mass ↑ MIP, MEP, knee-extension, and arm-flexion Quadriceps ↑ (13.1%) and biceps brachii ↑ (23.8%) MIP, MEP, and MVV ↑ MVV ↓ in CG 19.6% Gait speed and ASM—non-significant
Chang and Chiu, 2020 [26]	Non-randomized trial	Taiwan	123 older persons Six nursing facilities ≥60 years Living in long-term care institutions for >3 months Low levels of PA	IG: chair muscle strength training Sandbag or grip ball 50 min session Twice/week, 12 weeks	Body composition: BIA (sarcopenic obesity measure) Self-reported health (EQ-5D-3L) Physical and mental health (SF-36)	No significant changes in SMI or % Only time effect was observed No change in quality of life Anxiety/depression in IG ↓ 46.2%
Chun-De et al., 2017 [27]	Randomized controlled trial	Taiwan	46 women aged 67.3 (5.2) years Sarcopenic obesity Chronic conditions leading to physical limitations	IG: elastic resistance bands Degree of elasticity: yellow, red, green, blue, black, and silver 60 min (10 min warm-up, 30–40 min exercise, and 10 min cool down) Three sets, ten reps of concentric and eccentric contractions 12 weeks	Body composition (DEXA) Muscle strength (dynamometer) Muscle quality (strength: mass) Physical capacity (mobility tests—time up-go test, single leg balance, gait speed)	↑ fat-free mass (0.73 kg), leg lean mass (0.79 kg), ↓ absolute total fat mass (−1.25 kg), and % body fat (−1.83%) ↑ gait speed (+0.21 m/s), TUG (+1.42 s), single leg stance (+8.58 s) Relationship ↑ between leg lean mass and gait speed (r = 0.36; *p* < 0.05) At the end of 12 weeks, fewer in IG exhibit sarcopenia
Conlon et al., 2017 [28]	Randomized controlled trial	Australia	41 healthy, untrained older adults 65–81 years No associated chronic conditions	Three groups Non-periodized, block-periodized, and undulated 22 weeks, 3 days/week	Cross-sectional area Vertical jump Peak torque Isometric force Muscle activation patterns	All three groups ↑ pre–post outcomes No group differences No change in muscle activity or force development Periodization strategies may not improve outcomes
de Almeida et al., 2021 [29]	Randomized controlled trial	Brazil	Two studies Study 1: 15 obese older adults (age: 67.4 years; BMI: 35 kg/m^2^) Study 2: 16 obese older women (age: 65 years; BMI: 94 kg/m^2^)	Study 1 Three groups: (a) RT at 50% 1 RM, (b) RT at 70% 1 RM, and (c) control group 12 reps, 10 sets—knee extension exercise Study 2 Functional task exercise Two sets of 15 reps/set	Cognitive function Stroop Test Trail making test (TMT) maximum dynamic strength 1 RM	Study 1 TMT-A ↓ 50% 1-RM (ES = −0.62) and 70% 1-RM (ES= −0.48) but not after the control visit; similar trend in TMT-B While no change in Stroop A, Stroop B ↓ ES = −0.24 at 50% and ES = −0.32 at 70% 1-RM Study 2 TMT-A ↓ FE (ES = −0.32), but not the control group Similar trends were noted for Stroop Regardless of intensity, RT ↑ executive functions
de Sá Souza et al., 2022 [30]	Randomized controlled trial	Brazil	Community-dwelling Sao Paulo >65 years Sarcopenia after mass testing 28 with sarcopenia among volunteers	IG: 14, CG: 14 8 exercises, large muscle groups 3 sessions/week, 12 weeks Linear periodization model 1st week, 1 set of 50% 1 RM, 2nd week, 2 sets of 60% of 1 RM, 3rd to 12th weeks, 75% of 1 RM, 3 sets; inter-set rest—60–90 s	Strength: 1-RM Polysomnography Isokinetic/isometric dynamometer tests Hormone and inflammatory markers: TG, cholesterol, TNF, IGF-α, IL-6, and IL-10	Time to sleep onset (sleep latency) ↓ (16.09 ± 15.21 vs. 29.98 ± 16.09 min in RT vs. control group) Slow-wave sleep (N3 sleep) ↑ (0.70%, vs. −4.90%) Insomnia severity ↓ in the IG group Apnea/hour ↓ RT group Absolute and relative peak torque ↑ Interleukin-10 ↑ in RT group
Dinh et al., 2019 [31]	Randomized controlled trial	Belgium	Senior Project Intensive Training project 100 women Aged >65 years Living independently	Three groups: Strength training: 3 × 10 reps at 80% of 1 RM Strength endurance training: 2 × 30 reps at 40% 1 RM Control: passive stretching Six weeks	Before and after six weeks T-cell percentages and absolute blood counts Viral antibodies Cytomegalovirus serostatus—immunonephelometry	Only changes seen in strength endurance training *Cytomegalovirus sero+ve* ↓ senescence-prone T-cells ↑ CD8-naive T-cells ↑ 44% senescent-like T-cells/ 51% CD8+ *Cytomegalovirus sero-ve* No changes
Flor-Rufino et al., 2023 [32]	Randomized controlled trial	Spain	Fifty-one sarcopenic individuals Community-dwelling women Aged >70 years	IG (high-intensity RT) Twice weekly, 65 min, session in groups Six months/39 sessions Session: 10 min warm-up, a 45 min HIRT circuit, and a 10 min cool down Six strength exercises Three sets, 10–15 reps/set, 70% 1 RM CG to remain active	Sarcopenia status: peripheral and respiratory muscle Spirometry Respiratory muscle strength: MEP and MIP Before and after six months	Sarcopenia both peripheral and respiratory ↓ (50%) in IG CG ↓ FEV1 (Δ0.12 L) and ↓ FVC (Δ −0.18 L) IG no change in spirometry EQ-VAS ↑ in IG and ↓ in CG (Δ 73 points) Respiratory sarcopenia reverted in IG
Gadelha et al., 2016 [33]	Randomized controlled trial	Brazil	133 volunteers 60–80 years University neighborhood Absence of metabolic disorders	IG: 1 RM determination, 60% 1 RM progressed to 80% by the 16th week 24-week, thrice/week 75% attendance	Body composition (DEXA) Sarcopenic obesity index Isokinetic muscle torque—Biodex dynamometer	Fat-free mass↑ (0.60 kg) but ≈ fat mass in IG (−0.19kg) Sarcopenic index ↑ IG but ↓ CG Peak torque (Δ + 0.61 Nm) in CG and (12.42 Nm) in IG Appendicular fat-free mass (Δ + 0.29) in IG while −0.35 kgs in CG
Ghasemikaram et al., 2021 [34]	Randomized controlled trial (FROST)	Germany	Forty-three community-dwelling ♂ (72 years and older) Morphometric sarcopenia Osteopenia or osteoporosis in spine/hip	IG: single-set exercise training, twice per week, supervised Single set (8 ex) and double set (4 ex)/8–15 reps Supplements provided 16 months	Body composition (DEXA) Muscle and adipose tissue volume and fat fraction of the thigh (MRI)	Thigh and intra-fascia fat volume ↓ (−2%) in IG and ≈CG Intermuscular adipose tissue volume ↑ CG but ≈ EG Fat fraction ↑ 7.7% and ↓ 0.77% HIIRT is favorable for intramuscular adipose tissue and fascia fat
Heo and Jee, 2024 [10]	Randomized controlled trial	Korea	Seoul Seniors Tower residents Aged 65–75 years 81 participants (39 ♀ and 42 ♂)	Four groups: low (LIRT), moderate (MIRT), and high (HIRT) intensity; RT and CG 50 min/day, 3 days/week for 12 weeks Machine driven RT for large muscles Three sets, 2–3 min rest Progressed 5–10% 1-RM CG: meditation and stretching at the same time	Body composition: BIA CT 0 weeks and 12th week—thigh volumes Serum cytokine (IL-6, IL-10, TNF—α) and immune cells (CD4, CD8, NK)—flow cytometry	Moderate–high-intensity RT ↑ muscle mass IL-6 ↓ (−20.94%) in MIRT, TNF ↓ (−28.75%) in HIRT IL-10↑ (35.72%) in MIRT NK cells ↓ CG and ↑ IG (LIRT, MIRT, HIRT) CD3 and CD8 T ↑ in MIRT, HIRT Moderate–high-intensity RT—favorable anti-inflammatory effect
Kalapotharakos et al., 2010 [35]	Randomized controlled trial	Greece	47 community-dwelling men Aged >80 years Independent	Three groups (14 weeks) Supervised Resistance training (RT): 6 exercises, lower and upper muscle groups, 70% 1-RM, twice weekly, 14 weeks Resistance detraining (RDT): lower and upper muscle training for eight weeks, detraining for six weeks CG: no training for 14 weeks	8th and 14th weeks 6MWD, sit–stand, TUG test, and chair raise time	*RT and RDT at 8th week* 3-RM strength ↑—25% to 55% Functional performance ↑—15–25% *RDT at 14th week* Muscle strength ↓—60 to 87% Functional performance ↓—36 to 70% gains CG ≈ 8th and 14th weeks
Lai et al., 2021 [36]	Randomized controlled trial	China	60 pre-frail elderly individuals Hospitalized >60 years 17 ♂ and 13 ♀ in IG 15 ♂ and 15 ♀ in CG	12 weeks IG: sandbags (0.5–1 kgs) on ankles Back lifts, side lifts, knee bend—15 min CG: received routine care, face–face exercise advice	Lower limb muscle strength Physical fitness (Senior Fitness Test) Physical performance—6MWD, 30sSTS Energy metabolism (Actigraph wGT3X-BT3)	Quads femoris muscle strength ↑ in IG (Δ+2.3 kgs) compared to CG (−0.7 kgs) 6 MWD ↑ +111 m in IG compared to CG (−11 m) 30 s STS—↑ 4.4 times in IG while no change in CG Kcal ↑ +80 / 2MET↑ in IG
Nagai et al., 2018 [37]	Randomized controlled trial	Japan	Forty-one frail older adults Community-dwelling >65 years old Independent walk; no visual impairment	Six months Two IG: RPA—RT with PA, RT alone RT: twice weekly, 24 weeks, four upper and lower body RT exercises, progressed 50% to 80% of 1 RM PA advice: ↑ PA and step count and ↓ ST by 10% every week through automatic feedback system	Frailty status and frailty scores (gait speed, Dynamometer) Muscle strength (knee extension and leg press) Instrumental activities Quality of life Behavior change PA—wrist accelerometer (14 days)	RPA ↑ LIPA, daily steps, and lower-limb muscle strength and ↓ frailty (Δ −1.5 scores) compared to RT alone Knee extension (+1 vs. 0.3 kg/m), leg press (+19 kgs vs. 1.4 kg), MET (+1.8 vs. −0.1 MET hrs/day) There are no significant differences in instrumental activity or frailty status in the IG No change in quality of life or MVPA engagement
Perkin et al., 2019 [38]	Non-randomized trial	UK	Outpatients attending a memory clinic Twenty-one pre-frail outpatients Aged ≥65-years 3–4 score in short performance battery	Home-based “exercise snacking” Two groups: control (CG) and exercise snack (IG) group 28 days Twice daily Five muscle-strengthening exercises, each lasts for 1 min, and 1 min seated rest for a total of 9 min Functional RT exercises using body weight	Acceptability Knowledge and attitude towards RT Performance scores (SPPB), TUG, 60 s STS, and standing balance	80% adherence Intervention highly acceptable (4.6/5) A positive view of the intervention *Post 28 days vs. baseline* ↑ Short performance (8(1) vs. 9(3)) TUG (11.32 (4.02) vs. 9.18 (5.25) s) 60 s STS (17 ± 5 vs. 23 ± 7 reps) No difference in RPE and balance Single leg standing balance of left leg ↑ 11.27 vs. 20.33 s
Perreault et al., 2016 [9]	Non-randomized trial	Canada	26 sarcopenic men 60–75 years Appendicular mass index < 10.75 kg/m^2^ Inactive with no associated co-morbidities	16 weeks Thrice weekly one-hour session Large upper and lower body muscles Three sets, eight reps/set, 80% 1-RM Min 85% sessions	Body composition (DEXA)—appendicular mass index Serum biomarkers: eHSP72, hsIL-6, hs-CRP, and hsTNF-α (ELISA) Self-reported physical activity	At 4th month, weight, BMI, appendicular mass ↑ Sarcopenia scores ↓ eHSP72 ↓ (Δ −0.114 ng/mL) Concomitant ↑ LBM variables and appendicular muscle mass index No significant changes in serum hsIL-6, hs-CRP, or hsTNF-α Higher hsIL-6 is associated with lower muscle mass index
Rabelo et al., 2011 [39]	Randomized controlled trial	Brazil	154 elderly women 60–86 years Sedentary > 6 months Seventy-eight volunteers completed	Two groups: RT and CG RT: 3 times/week; progression: 60% of 1 RM in 1st 4 weeks, 70% 4–8 weeks, and 80% rest of the 16 weeks, three sets Machines with plates: chest press, lateral pulldown, knee extension, hamstring curl, leg press, hip abduction Reps ↓ 12, 10, and 8/24 weeks	Dominant knee extension peak torque (isokinetic dynamometer) FFM (DEXA) Appendicular FFM—Arms FFM + Legs FFM	*RT vs. CG* Knee extensor peak torque ↑ (+14 Nm—15.6%) FFM ↑ (0.7 kg) Appendicular FFM ↑ (0.3 kg) 1 RM values ↑ 33.1% (lateral pull down) to 70% (bench press)
Ramirez-Campillo et al., 2018 [40]	Randomized controlled trial	Chile	74 older women 52 for final analyses Hispanic	High-speed training: bench press, row, biceps and leg curl, leg press, medicine ball throwing, countermovement jump, back and abdominal extensors 60 min, three times per week, 45%, 60%, and 75% of their baseline 1 RM, 8 reps/set IG: traditional and cluster (metronome) rest between sets for 150 s for traditional while 30 s for cluster 12 weeks	10 m walking speed test 8 foot up-and-go test Sit-to-stand test Physical quality of life (menopause-specific quality of life questionnaire)	Both intervention groups ↑ outcomes Cluster: ↑ 10 m walking speed test, 8-TUG test, sit-to-stand test, and quality of life Traditional: 10 m walking speed test, 8-TUG test, and sit-to-stand test No change in CG Both training groups are equally effective
Ribeiro et al., 2022 [41]	Non-randomized controlled trial	Brazil	Thirty older women 60 years old or older Physically independent Non-hypertensive	LOW: estimated load at 15 RM MOD: estimated load at 10 RM Eight weeks Major muscles of upper and lower limbs	Body composition (DEXA) Maximal dynamic strength—1-RM Muscular quality index (sum of 1-RM thrice/muscle mass) 1–2 weeks and 11–12 weeks	Results similar (LOW = MOD) LOW load (15 RM) ≈ MOD load (10 RM) in ↑ muscle quality and fat-free mass
Saeterbakken et al., 2018 [42]	Non-randomized trial	Norway	30 older adults Aged >70 years Home-based	Progressive RT Twice a week 10–12 repetitions for ten weeks. Initially, two sets progressed to 3 sets >80% of assigned sessions Squats, box lifts, seated rows, chest press, and biceps curls	Strength (maximal and rate of force development) Functional test (walk, chair squat time, and distance) Accelerometer-measured PA	23 completed the study No change in strength or force development, physical function, or physical activity at the end of the 10th week Twice a week with a low load did not alter the physical function or strength
Schulte and Yarasheski, 2001 [43]	Non-randomized trial	USA	Short term: 7 healthy young and 7 healthy older men and women Long term: 17 old adults with frailty	Short-term (2 weeks): 10 RT sessions Prolonged exercise: stretching and flexibility and low-intensity RT sessions	MVC (1-RM) Isokinetic and isometric torque—knee extensors Biochemical sample—leucine—myosin heavy chain	Short-term ↑ mixed muscle protein synthetic rates (0.05 to 0.1%/hr) in vastus lateralis Prolonged RT ↑ myosin heavy chain synthesis and mixed muscle proteins (100 to 140 mg/kg/hr) Serum myostatin–immunoreactive protein levels have an inverse correlation with lean mass
Seo et al., 2021 [44]	Non-randomized controlled trial	South Korea	27 (22 completed) older adult women Aged >65 years with sarcopenia	IG: thrice a week, 60 min per session for 16 weeks 5 min warm-up, 50 min RT, and 5 min cool down Five RT ex (squat, split squat, push up, back extension, knee to chest)	Body composition and thigh composition (DEXA and CT) Isometric muscle strength (isokinetic dynamometer) Muscle growth factors (growth factors, follistatin)	WHR ↓ (F = 7.19, η2p = 0.264) IG: ↑ physical fitness, gait, handgrip IG: ↑ growth factors include follistatin but not others ↑ fitness and ↓ age-related ↑ in thigh intramuscular fat
Silva et al., 2023 [45]	Non-randomized trial	Brazil	74 participants >60 years/no physical limitations CG (n = 37) and IG (n = 37). Excluded if attended <70% of sessions	12 weeks of RT Three times a week Initial 60% 1 RM, 12–15 reps Final 85% 1 RM, 6–8 reps	Strength (handgrip) Muscle mass (bioimpedance) Physical performance tests (chair stand, SPPB, and walk)	1 RM ↑ 10 kgs after 12 weeks in IG ↑ TUG and five second sit to stand ↑ handgrip (IG +2 kgf, CG −7kgf) No change in muscle mass index SBP ↓ (−25 mmHg) after 12 weeks in IG
Stoever et al., 2018 [46]	Non-randomized controlled trial	Germany	55 physically inactive Obese (BMI ≥ 30 kg/m^2^) Older adults (≥65 years) Without severe disease	*Sarcopenia group* Progressive RT Initially: 60% of max. strength Two sets of 12 to 15 reps 60 min/session Twice/week, 16 weeks 4–8 weeks ↑ 80–85% of maximum strength Three sets of 8–12 reps	Body composition SPPB—physical functions Handgrip strength Muscle mass index Functional Reach Test	Sarcopenia group, handgrip strength (+9%), gait speed (+5%), SPPB score (+13%), and modified PPT score (+11%). Physical performance reaches non-sarcopenic baseline Non-sarcopenic: ↑ SPPB +10%, modified PPT score+7% There was no change in SMI or the functional reach test
Van Roie et al., 2013 [16]	Non-randomized controlled trial	Belgium	56 community-dwelling adults Aged 60 and older No risk of chronic diseases	Three interventions Leg press and leg extension training High- and low-volume reps 12 weeks, 36 sessions	1 RM of leg presses and extensions Lower body muscle mass—CT scan Knee extensors—peak torque Dynamic peak torque at varying speeds Functional performance test	Adherence is better with high volume High volume ↑ dynamic peak torque at 240º ↑ 1 RM strength No changes in speed Leg press 1 RM ↑ high volume 46.2%, low volume + 39.2% High volume—maximal gait speed and gait speed ↑ High and low fatigue failure improves hypertrophy
Vezzoli et al., 2019 [1]	Randomized controlled trial	Italy	35 sarcopenic elderly individuals Over 65 years Independent Community-dwelling Absence of chronic conditions	IG: 20, thrice/week, 12 weeks 6–8 min warm-up 3 sets, 14–16 reps Upper and lower body RT with free weights at 60% 1 RM	Body composition Short physical performance battery Handgrip TUG and stair climbing tests. Ultrasound—muscle morphology (vastus lateralis, rectus femoris) ROS production	Sarcopenia ↓ 15% compared to pre-training There is no difference in skeletal muscle mass, SPPB score, handgrip, or get-up-and-go tests IG ↑ stair climbing by 7.7% IG ↑ VL—5.5%, EF: 10.4%, RF: 14.5% 1 RM ↑ 66.7% to 101% ROS ↓ (−21.2%) Markers of oxidative stress ↓
Vikberg et al., 2019 [15]	Randomized controlled, parallel-group, 2-arm trial	Sweden	70 selected from the Healthy Ageing Initiative Northern Sweden Appendicular lean mass index < 7.29 in men and ≤5.93 in women	Progressive RT Three sessions (∼45 min each) per week Ten weeks RPE = 6–7 8 exercises targeting large muscles	Primary: SPPB score Secondary: TUG test, chair STS, isometric muscle strength, lean body mass, and fat mass	No significant effect on SPPB Male ↑ 0.5 points in SPPB IG ↓ chair sit–stand time by 0.9 ± 0.6 s Lean body mass ↑ 1147 g and total fat mass ↓ 553 ± 225 g in IG
Yuenyongchaiwat et al., 2023 [14]	Randomized controlled trial	Thailand	32–60 years 90 elderly individuals (>60 years) 60 with sarcopenia (IG 30, CG 30) and without sarcopenia	Pedometer ↑ walking—7500 steps/day, five days/week + RT with an elastic band Twice/week, 12 weeks	Inflammatory profiles (IL-6, TNF-α Depression scale	IG: 2142 avg steps/day baseline, 7575 steps/day at the end of 12 weeks Depressive symptoms, IL-6, and TNF- α ↓ without sarcopenia There are no significant differences between CG and IG with sarcopenia

Abbreviations: 1-RM—one repetition maximum, BMC—bone mineral content, BMI—body mass index, BP—blood pressure, CG—control group, COP—center of pressure, CRP—C-reactive protein, DEXA—dual energy X-ray absorptiometry, EMG—electromyography, ES—effect size, FEV—force expiratory volume, FFA—free fatty acid, HIIRT—high-intensity interval resistance training, HSP—heat shock proteins, IG—intervention group, IGF—insulin like growth factor, IL—interleukin, IPAQ—international physical activity questionnaire, LIPA—light-intensity physical activity, MEP—maximal expiratory pressure, MET—metabolic equivalent, MIP—maximal expiratory pressure, MRI—magnetic resonance imaging, MVPA—moderate to vigorous physical activity, MVV—maximal voluntary ventilation, MWD—minute walk distance, PA—physical activity, PRT—progressive resistance training, RNA—ribonucleic acid, RM—repetition maximum, ROS—reactive oxygen species, RPE—rate of perceived exertion, RT—resistance training, SBP—systolic blood pressure, SMI—skeletal muscle index, SPPB—short physical performance battery, STS—sit to stand, TG—triglycerides, TMT—trail making test, TNF—tissue necrosis factor, TUG—Time-Up-Go test, USA—United States of America, VAT—visceral adipose tissue, VO2—oxygen consumed for the workload. ↑ denotes ‘increase’; ↓ denotes ‘decrease’.

**Table 3:** Dose of RT programs employed in studies to counter sarcopenia in healthy older adults.

**Dose of the RT Program**	**Site of Training**	
	**Access to Traditional Gyms**	**Only Home-Based Programs**
Type	Circuit training, progressive	Conventional, progressive
Equipment	Machine plates, barbells with incremental weights	Body weights, TheraBands, medicine balls, TRX
Intensity	60–85% of 1-RM, 60–90% of maximal voluntary contraction	Not specific, sometimes based on progressive elastic resistance (different colors)
Volume	8–15 reps/set, 2–3 sets/muscle, eight larger muscles	
Progression	1st two weeks 55–65% 1-RM 12–15 reps, two sets, 3–4 weeks, 65–75% 1-RM, 2–3 sets, 10–12 reps, 5–6 weeks 75–85% 1-RM 8–10 reps/set, 6–8 reps/set, three sets at 6–8 weeks. In the 8th week, new 1-RM test	1st two weeks, 12–15 reps, two sets progressing to 6–8 reps, three sets at 6–8 weeks, thereby progressing the number of reps and sets as per the individual’s ability
Duration per single session	30–50 min with at least 5 min of warm-up and cool down with stretches	
Duration for clinically meaningful change	Eight weeks (6 weeks to 1 year)	12 weeks (10 weeks to 2 years)
Adjunct	Balance and flexibility: Tai-Chi and Yoga	Balance exercises—body support exercises
Nutritional supplements	Protein supplements (reinforcing protein synthesis)	Dearth of evidence
Group exercise	Not possible	A group of 6–8 members, chair-based or traditional group exercises

Abbreviations: reps—repetitions, 1-RM—one repetition maximum, RT—resistance training.

## Data Availability

No new data were created or analyzed in this study. Data sharing is not applicable to this article.

## Supplementary Materials

The following supporting information can be downloaded at https://www.mdpi.com/article/10.3390/life15050688/s1, Supplementary File S1: Preferred Reporting Items for Systematic reviews and Meta-Analyses extension for Scoping Reviews (PRISMA-ScR) Checklist; Supplementary File S2: MeSH term combination and search strategy.

## Abbreviations

The following abbreviations are used in this manuscript:

BMC	bone mineral content
BMI	body mass index
BP	blood pressure
CG	control group
COP	center of pressure
CRP	C-reactive protein
DEXA	dual energy X-ray absorptiometry
EMG	electromyography
FEV	force expiratory volume
FFA	free fatty acid
HIIRT	high-intensity interval resistance training
HSP	heat shock proteins
IGF	insulin like growth factor
IL	interleukin
IPAQ	international physical activity questionnaire
LIPA	light-intensity physical activity
MEP	maximal expiratory pressure
MET	metabolic equivalent
MIP	maximal expiratory pressure
MVPA	moderate to vigorous physical activity
MVV	maximal voluntary ventilation
MWD	minute walk distance
PA	physical activity
PRT	progressive resistance training
RM	repetition maximum
ROS	reactive oxygen species
RPE	rate of perceived exertion
RT	resistance training
SBP	systolic blood pressure
SMI	skeletal muscle index
SPPB	short physical performance battery
STS	sit to stand
TG	triglycerides
TMT	trail making test
TNF	tissue necrosis factor
TUG	Time-Up-Go test
VAT	visceral adipose tissue
VO2	oxygen consumed for the workload
