# Supplementary material for: Effects of Resistance Training on Sarcopenia Risk Among Healthy Older Adults: A Scoping Review of Physiological Mechanisms
Source: Life (Basel). 2025 Apr 23;15(5):688. doi: 10.3390/life15050688 (PMC12112962; doi:10.3390/life15050688)
Supplement: Supplementary file 1 [file life-15-00688-s001.zip › life-3554740-supplementary.pdf]

Resistance Exercise Training for Mitigating Sarcopenia in Healthy Older Adults: A Scoping Review of Physiological Mechanisms

Supplementary files

Supplementary file S1. PRSIMA-ScR checklist

Supplementary file S2. MeSH term combination and search strategy

Supplementary file S1. PreferredReportingItemsforSystematicreviewsandMeta-Analysesextensionfor Scoping Reviews (PRISMA-ScR) Checklist

SECTION	ITEM	PRISMA-ScRCHECKLISTITEM	REPORTED ONPAGE#
**TITLE**			
Title	1	Identifythereportasascopingreview.	1
**ABSTRACT**			
Structured summary	2	Provide a structured summary that includes (as applicable):background,objectives,eligibilitycriteria, sources of evidence, charting methods, results, and conclusionsthatrelatetothereviewquestionsand objectives.	1
**INTRODUCTION**			
Rationale	3	Describetherationaleforthereviewinthecontextof what is already known. Explain why the review questions/objectives lend themselves to a scoping review approach.	2
Objectives	4	Provide an explicit statement of the questions and objectives being addressed with reference to their key elements(e.g.,populationorparticipants,concepts,and context) or other relevant key elements used to conceptualizethereviewquestionsand/orobjectives.	2
**METHODS**			
Protocoland registration	5	Indicate whether a review protocol exists; state if and where it can be accessed (e.g., aWeb address); and if available,provideregistrationinformation,includingthe registration number.	NA
Eligibilitycriteria	6	Specifycharacteristicsofthesourcesofevidenceused as eligibility criteria (e.g., years considered, language, andpublicationstatus),andprovidearationale.	3
Information sources*	7	Describe all information sources in the search (e.g., databases with dates of coverage and contact with authorstoidentifyadditionalsources),aswellasthe date the most recent search was executed.	3
Search	8	Presentthefullelectronicsearchstrategyforatleast1 database,includinganylimitsused,suchthatitcouldbe repeated.	3
Selectionof sources of evidence†	9	Statetheprocessforselectingsourcesofevidence(i.e., screening and eligibility) included in the scoping review.	3
Datacharting process‡	10	Describethemethodsofchartingdatafromtheincluded sourcesofevidence(e.g.,calibratedformsorformsthat have been tested by the team before their use, and whether data charting was done independently or in duplicate)andanyprocessesforobtainingand confirming data from investigators.	4
Dataitems	11	Listanddefineallvariablesforwhichdataweresought and any assumptions and simplifications made.	4
Criticalappraisalof individual sources of evidence§	12	If done, provide a rationale for conducting a critical appraisal of included sources of evidence; describe the methodsusedandhowthisinformationwasusedinany data synthesis (if appropriate).	4
Synthesisofresults	13	Describethemethodsofhandlingandsummarizingthe data that were charted.	4

SECTION	ITEM	PRISMA-ScRCHECKLISTITEM	REPORTED ONPAGE#
**RESULTS**			
Selectionof sources of evidence	14	Give numbers of sources of evidence screened, assessed for eligibility, and included in the review, with reasonsforexclusionsateachstage,ideallyusingaflow diagram.	4-5
Characteristicsof sources of evidence	15	Foreachsourceofevidence,presentcharacteristicsfor which data were charted and provide the citations.	5-19
Criticalappraisal withinsourcesof evidence	16	Ifdone,presentdataoncriticalappraisalofincluded sources of evidence (see item 12).	Table 1,
Resultsof individualsources of evidence	17	Foreachincludedsourceofevidence,presentthe relevantdatathatwerechartedthatrelatetothereview questions and objectives.	20-21
Synthesisofresults	18	Summarizeand/orpresentthechartingresultsasthey relate to the review questions and objectives.	20-21
**DISCUSSION**			
Summaryof evidence	19	Summarize the main results (including an overview of concepts,themes,andtypesofevidenceavailable),link tothereviewquestionsandobjectives,andconsiderthe relevance to key groups.	21-23
Limitations	20	Discussthelimitationsofthescopingreviewprocess.	23
Conclusions	21	Provide a general interpretation of the results with respecttothereviewquestionsandobjectives,aswell aspotentialimplicationsand/ornextsteps.	23
**FUNDING**			
Funding	22	Describesourcesoffundingfortheincludedsourcesof evidence,as wellassourcesof fundingforthescoping review. Describe the role of the funders of the scoping review.	24

Supplementary file S2. MeSH term combination and search strategy

“Strength training” OR “resistance exercise” OR “resistance exercise training” OR dumbbell OR “barbell training” OR “kettle bell” OR “weight training” OR “callisthenics” OR “resistance bands”

AND

Sarcopenia OR “muscle loss” OR dynapenia OR frailty

Search strategy and results

Ebsco = 735

(strength training or resistance training or weight training) AND (sarcopenia or sarcopenic or muscle weakness or muscular atrophy or muscle loss)

Embase = 1724

('resistance exercise training'/exp OR 'resistance exercise training' OR 'strength training'/exp OR 'strength training' OR dumbell OR 'weight lifting'/exp OR 'weight lifting' OR calisthenic OR 'body supported training' OR 'resistance training'/exp OR 'resistance training' OR 'resistance exercise'/exp OR 'resistance exercise') AND (sarcopenia:ti,ab,kw OR 'sarcopenic obesity':ti,ab,kw OR 'sarcopenia index':ti,ab,kw)

Web of Science = 1769

https://www.webofscience.com/wos/woscc/summary/fea4f7cf-1139-419d-b8ca-feaf5d0f8e98-e333130b/relevance/1

“Strength training” OR “resistance exercise” OR “resistance exercise training” OR dumbbell OR “barbell training” OR “kettle bell” OR “weight training” OR “calisthenic” OR “resistance bands” (All Fields) and Sarcopenia OR “muscle loss” OR dynapenic OR frailty

Scopus = 1138

(TITLE-ABS-KEY ("strength training" OR "resistance exercise" OR "resistance exercise training" OR dumbbell OR "barbell training" OR "kettle bell" OR "weight training" OR "calisthenic" OR "resistance bands") AND TITLE-ABS-KEY (sarcopenia OR "muscle loss" OR dynapenic OR frailty))
